# Relationship of hemoglobin levels with outcomes in deceased donor kidney transplant: a retrospective cohort study

**DOI:** 10.1590/2175-8239-JBN-2023-0014en

**Published:** 2024-01-26

**Authors:** Beatriz Moreira Silva, Flavia Hosana Macedo, Enzo Eiji Miyasato Hayano, Suzeli Germano, Isabella Ferreira Ribeiro, Carolina Azze Franco, Lucio Requião, José Medina-Pestana, Miguel Angelo Goes

**Affiliations:** 1 Universidade Federal de São Paulo, Divisão de Nefrologia, São Paulo, SP, Brazil.; 2 Universidade Federal de São Paulo, Departamento de Medicina, São Paulo, SP, Brazil.; 3 Universidade Federal de São Paulo, Hospital do Rim, São Paulo, SP, Brazil.

**Keywords:** Anemia, Deceased Donor, Kidney Transplantation, Delayed Allograft Function, Chronic Kidney Allograft Dysfunction, Anemia, Doador Falecido, Transplante de Rim, Função Retardada do Aloenxerto, Disfunção Crônica do Enxerto Renal

## Abstract

**Introduction:**

Anemia is frequent in patients undergoing replacement therapy for kidney failure. Anemia in the pre- and post-transplantation period might be related to kidney transplant outcomes. The current study therefore sought to assess the relationship between anemia, delayed allograft function (DGF), chronic kidney allograft dysfunction (CAD), and death from any cause following kidney transplantation from a deceased donor.

**Methods:**

This was a retrospective study with 206 kidney transplant patients of deceased donors. We analyzed deceased donors’ and kidney transplant patients’ demographic data. Moreover, we compared biochemical parameters, anemia status, and medicines between DGF and non-DGF groups. Afterward, we performed a multivariate analysis. We also evaluated outcomes, such as CAD within one year and death in ten years.

**Results:**

We observed a lower frequency of pre-transplant hemoglobin concentration (Hb) but higher frequency of donor-serum creatinine and red blood transfusion within one week after transplantation in the group with DGF. In addition, there was an independent association between Hb concentration before transplantation and DGF [OR 0.252, 95%CI: 0.159–0.401; p < 0.001]. There was also an association between Hb concentration after six months of kidney transplantation and both CAD [OR 0.798, 95% CI: 0.687–0.926; p = 0.003] and death from any cause.

**Conclusion:**

An association was found between pre-transplantation anemia and DGF and between anemia six months after transplantation and both CAD and death by any cause. Thus, anemia before or after transplantation affects the outcomes for patients who have undergone kidney transplantation from a deceased donor.

## RESUMO

**Introdução:**

A anemia é frequente em pacientes submetidos à terapia substitutiva para insuficiência renal. A anemia nos períodos pré e pós-transplante pode estar relacionada aos desfechos do transplante renal. Portanto, o presente estudo buscou avaliar a relação entre anemia, função retardada do enxerto (FRE), disfunção crônica do enxerto renal (DCE) e óbito por qualquer causa após transplante renal de doador falecido.

**Métodos:**

Este foi um estudo retrospectivo com 206 pacientes transplantados renais de doadores falecidos. Analisamos dados demográficos de doadores falecidos e pacientes transplantados renais. Além disso, comparamos parâmetros bioquímicos, status de anemia e medicamentos entre os grupos FRE e não-FRE. Posteriormente, realizamos uma análise multivariada. Também avaliamos desfechos, como DCE em um ano e óbito em dez anos.

**Resultados:**

Observamos menor frequência de concentração de hemoglobina (Hb) pré-transplante, mas maior frequência de creatinina sérica do doador e transfusão de hemácias no período de uma semana após o transplante no grupo FRE. Além disso, houve associação independente entre a concentração de Hb antes do transplante e a FRE [OR 0,252; IC 95%: 0,159–0,401; p < 0,001]. Houve também associação entre a concentração de Hb após seis meses de transplante renal e ambos, DCE [OR 0,798; IC95%: 0,687–0,926; p = 0,003] e óbito por qualquer causa.

**Conclusão:**

Encontrou-se uma associação entre anemia pré-transplante e FRE e entre anemia seis meses após o transplante e ambos, DCE e óbito por qualquer causa. Assim, a anemia antes ou após o transplante afeta os desfechos de pacientes que foram submetidos a transplante renal de doador falecido.

## Introduction

Anemia and cardiovascular disease are frequent and related to complications in patients with kidney failure (KF) on replacement therapy (RT). Cardiovascular disease is the leading cause of death in patients on KFRT^1,2^. Anemia in KFRT patients is multifactorial but primarily caused by insufficient erythropoietin (EPO) production^3^. Other factors related to decreased GFR, such as iron deficiency, oxidative stress, inflammation, uremic solutes, and toxins, might contribute to CKD-related anemia^2,3,4,5,6^.

Therefore, most patients with KFRT require an erythropoiesis-stimulating agent (ESA). Others still need red blood cell transfusion for anemia treatment^7,8^. Kidney transplantation can restore kidney function, including EPO production. Therefore, kidney transplantation can increase survival and life quality^8^. Delayed allograft function (DGF) is associated with cold ischemia time and other clinical factors^5,6^. DGF is the clinical diagnosis when the patient still needs dialysis within the first week after KT, adversely impacting short- and long-term allograft survival^6,7^.

Kidney chronic allograft dysfunction (CAD) is a clinical entity defined as a slowly rising creatinine owing to progressive decreased kidney function with associated hypertension and proteinuria^8^. CAD is associated with DGF, acute rejection, and anemia^8–11^. Moreover, it is characterized by morphological deterioration, occurring at least 3–6 months and usually one year after transplantation in the absence of active acute rejection, drug toxicity, renal vascular disease, or other kidney diseases^8–10^. Such allograft changes require percutaneous renal biopsy when there is unexplained proteinuria or serum creatinine increase. Moreover, CAD is the most critical kidneys disease in the long-term after kidney transplantation and is an established dysfunctional histopathological condition. It may present with intimal thickening, glomerular capillary wall thickening, chronic interstitial fibrosis, and tubular atrophy in the renal allograft. CAD has no specific therapy and is mediated by several mechanisms, but requires supportive therapy^9,10^.

In addition, anemia can occur after kidney transplantation, with a higher prevalence just after transplantation^11,12^. Leading causes of anemia in the early post-transplant period include surgery-related blood loss, malnutrition, medications, and overhydration, immunosuppressive drugs, inflammation, inadequate production, and resistance to erythropoietin (EPO)^13–18,19^. Therefore, we verified whether pre- and post-transplant hemoglobin concentrations (Hb) are associated with DGF, CAD, and death from any cause in deceased donor kidney transplant recipients.

## Methods

### Study Design and Patients

This was a retrospective observational cohort study that included all patients that received a deceased donor kidney transplant between January 1, 2008, and December 31, 2008, that fulfilled the inclusion criteria: age ≥18 years, receiving an organ from a donor with creatinine ≤ 5.0 mg/dL irrespective of age. Exclusion criteria were: undergoing peritoneal dialysis for the last two months prior to transplantation, pregnant women, multiple-organ transplant, having an advanced neurological disease or a psychiatric condition, those lost to follow-up, deceased within 28 days of transplantation, and not attaining or not maintaining adequate calcineurin inhibitors serum levels. Censoring occurred at the date of death, graft loss, or end of follow-up.

The study verified possible associations between pre-transplant, immediate post-transplant, and six months post-transplant hemoglobin levels and DGF, CAD, and death by any cause.

All kidney transplant recipients received immunosuppression induction with 1 g of methylprednisolone intraoperatively and thymoglobulin in the immediate postoperative period for 2 ± 1 days.

The study was approved by the Research Ethics Committee of the Federal University of São Paulo (30942614.9.0000.5505/2014) and carried out under specific national legislation, the recommendations of the local Research Ethics Committee, the guidelines of the Declaration from Helsinki, and the Declaration of Istanbul^20,21^. The informed consent was waived due to the retrospective observational nature and the information being anonymized and de-identified.

### Variables of Interest

For recipients, the variables of interest were age, sex, comorbidities, chronic kidney disease (CKD) etiology, and the following baseline data: mean arterial pressure and use of antihypertensive agents; blood glucose level; serum creatinine; parathyroid hormone, ionic calcium levels, Hb concentration, iron status, red blood cell transfusion record, medicines; use of erythropoiesis-stimulating agents (ESA), and iron supplementation. Donors were separated into standard donors and expanded criteria donors. For deceased donors, the variables were age, final serum creatinine level, cold ischemia time (CIT), Kidney Donor Risk Index (KDRI), Kidney Donor Profile Index (KDPI)^22,23,24,25,26,27^, and type of donor stratified into standard or expanded criteria^28^.

The pre-transplant variables were: sex, age, diabetes mellitus, hypertension, mean arterial pressure (MAP), creatinine, hemoglobin, serum iron, transferrin saturation, ferritin, leukocyte, and platelet. After transplantation, all patients in the study followed the 2008 institutional protocol for immunosuppression of deceased donors recipients with intravenous methylprednisolone and thymoglobulin. Maintenance therapy for these recipients involved three classes of oral immunosuppressive agents, prednisone as a corticosteroid, associated with an antimetabolite agent, such as azathioprine or mycophenolate. The third immunosuppressive component consisted of a calcineurin inhibitor, cyclosporine, or tacrolimus, which has potential nephrotoxicity. Thus, the nephrologist’s team carefully evaluated the serum level maintenance target for calcineurin inhibitors after kidney transplantation from deceased donors with expanded criteria or acute kidney injury. The maintenance target serum levels for cyclosporine is between 100 and 300 ng/mL and for tacrolimus is between 8 to 12 ng/mL. All expanded criteria deceased donor kidney recipients had tacrolimus introduced late to minimize risks of nephrotoxicity influence. We considered the following variables: time under induction therapy (in days), requirement for dialysis or red blood transfusion within seven days, and acute rejection episodes. We also analyzed the serum creatinine of the DGF group (3 months; 91 ± 12 days) and non-DGF group (3 months, 92 ± 11 days), Hb concentration, and serum creatinine after kidney transplantation of the DGF group (6 months; 184 ± 19 days) and of the non-DGF group (6 months; 179 ± 15 days).

Then, the CKD-EPI equation was used to estimate kidney function^29,30,31^. Moreover, a 15 to 20% increase in serum creatinine from baseline suggests kidney graft dysfunction, warranting ultrasound evaluation of the allograft and possibly a kidney allograft biopsy by hospital institution protocol^32^.

### Outcomes

We analyzed three different outcomes at three time points: DGF within one week after transplantation, CAD within one year after transplantation, and death for any cause within ten years after kidney transplantation.

### Definitions

We considered DGF when patients with deceased kidney transplantation need dialysis in the first seven days after KT^9,10^. CAD was defined as a progressive kidney dysfunction with morphological alteration in a renal allograft biopsy occurring within 3–12 months after transplantation in the absence of active acute rejection, drug toxicity, or renal vascular disease^15,16,17^. Even so, we defined acute rejection when serum creatinine increased that was confirmed with a kidney graft biopsy^24^. Lastly, anemia was defined as a hemoglobin level <13.0 g/dL for men and postmenopausal women or <12.0 g/dL for premenopausal women^3,33,34,35,36^.

Extended criteria donor (ECD) kidneys are kidneys from donors aged ≥60 or donors aged ≥50 who meet at least two of the following conditions: serum creatinine >1.5, death due to cerebrovascular accident, or history of hypertension. We used two indices to assess the deceased donor profile. Kidneys with KDPI scores ≥85 share similar donor traits with ECD kidneys, and KDPIs ≤60 are categorized as low-risk and high-quality^37^. The KDRI is a tool for assessing graft survival after transplants^38^. Chronic kidney disease (CKD) is characterized by either kidney damage or an estimated glomerular filtration rate (eGFR) below 60 mL/min/1.73 m^2^ persisting for three months or more, regardless of the underlying cause^39^.

The study verified possible associations of pre-transplant, immediate post-transplant, and six months post-transplant hemoglobin levels with DGF, CAD, and death by any cause.

Finally, the patients followed the institution’s immunosuppressive protocol.

### Statistical Analyses

Continuous variables with normal distribution are reported as mean ± SD, while non-normally distributed variables were submitted to logarithmic conversion. The one-sample Kolmogorov-Smirnov test ascertained the normality of distributions.

Categorical data are reported as percentages. We performed Pearson’s correlation for two variables. Next, we stratified the patients into DGF and non-DGF according to the diagnosis. The categorical data were compared by chi-square or Fisher’s exact test. After that, the variables associated with the probability of DGF were included in the multivariate analyses by logistic regression, with the backward deletion of predictive variables. All variables presenting a p-value ≤ 0.10 in the univariate analysis were included in the multivariate modeling.

Differences were considered statistically significant when two-tailed tests yielded a p < 0.05. The SPSS statistical software program (version 21.0; SPSS Inc., Chicago, IL, USA) was used for all statistical analyses.

## Results

**Figure 1.**
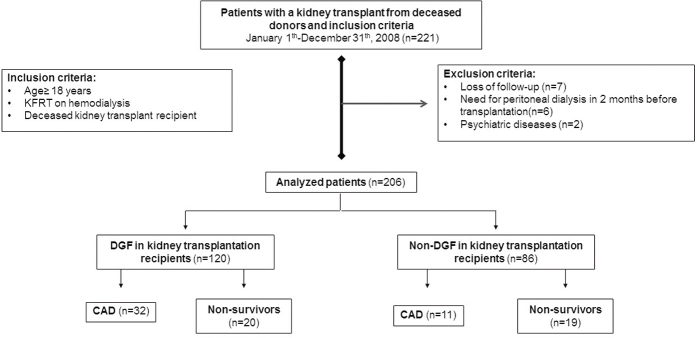
Flowchart diagram of the participants. KFRT: kidney failure on replacement therapy; DG:, delayed allograft function; non-DGF: non-delayed kidney allograft function; CAD: chronic graft dysfunction.

It analyzed two hundred and six patients based on the inclusion and exclusion criteria (Figure 1). Of the 206 patients, twenty-five patients (12.1%) had expanded donors. All study patients with deceased donor kidney transplantation for maintenance immunosuppression used calcineurin inhibitors and corticosteroids. Two patients in the present study were not using antiproliferative drugs. Even so, 204 kidney transplant patients in the study used either azathioprine or mycophenolate. In addition, we observed that 120 patients (58.2%) developed DGF. Thus, it was possible to compare two groups, DGF and non-DGF. Kidney donation from an expanded donor was observed in 9 patients (10.5%) in the non-DGF group and 16 patients (13.3%; p = 0.53) in the DGF group. The mean time from blood collection to assess serum creatinine after three months of kidney transplantation was 92+11 days in the non-DGF group and 91 + 12 days in the DGF group (p = 0.37). While the blood sample to analyze the Hb and serum creatinine concentration after six months of kidney transplantation was 179 ± 15 days for the non-DGF group and 184 ± 19 days for the DGF group (p = 0.08).

All patients in the DGF group required hemodialysis in the first week after kidney transplantation. The duration of need for hemodialysis after kidney transplantation was 10.5 ± 5.3 days. In the DGF group, five patients (4.2%) had undergone kidney transplantation previously, while three patients (3.5%) in the non-DGF group had previously undergone kidney transplantation (p = 0.80). We did not observe differences in serum ionic calcium (1.34 ± 0.50, 1.29 ± 0.11; p = 0.46) or parathyroid hormone (496 ± 59.4, 405 ± 71.4; p = 0.33) levels between DGF and non-DGF groups, respectively, at baseline.

Patients used an erythropoiesis-stimulating agent with the last dose within 72 hours before kidney transplantation (107 [89.2%], 72 [83.7%]; p = 0.25; on the DGF and non-DGF groups, respectively). All patients using an erythropoiesis-stimulating agent also used iron supplementation. One patient in each DGF and non-DGF group did not use antiproliferative drugs. Thus, we observed some patients receiving azathioprine (34 [28.3%]; 41[47.7%]; p = 0.02), while there were some patients in the use of mycophenolate (85 [70,8%]; 44 [51,2%], p = 0.02, on the DGF and non-DGF groups, respectively).

Deceased donors were older and had higher serum creatinine for the DGF group than in the non-DGF group (Table 1). There was a lower Hb concentration in the pre-transplant period in the DGF group (Table 2). Diabetes mellitus was the leading cause of KF (47 [39.2%]; 34 [9.5%], p = 0.41). Hypertension was the cause of KF (23 [19.2%];16 [18.6%]; p = 0.44). We found no difference in serum PTH levels between DGF (495 + 59.4 pg/mL) and non-DGF (405 + 71.4 pg/mL; p = 0.33) groups (Table 3).

**Table 1.** Donor characteristics and recipient early outcome (DGF)

	DGF (n = 120)	Non-DGF (n = 86)	p
**Expanded donor (%)**	16 (13.3)	9 (10.5)	0.53
**Donor age (years)**	45 ± 13	42 ± 15	0.08
**Donor serum creatinine (mg/d/L)^#^**	1.7 ± 0.67	1.4 ± 0.42	<0.001
**CIT (h)**	27.1 ± 6.2	27.1 ± 17.1	0.99
**KDPI**	58.6 ± 25.5	52.2 ± 29.8	0.10
**KDRI**	1.15 ± 0.35	1.12 ± 0.39	0.49

Data are reported as number (percentage) or mean and SD. DGF: delayed graft function; ^#^logarithmic transformation for statistical analysis; CIT: cold ischemia time; KDP, kidney donor profile index; KDRI: kidney donor risk index.

**Table 2.** Binary logistic regression with kidney delayed graft function as the response variable and its predictors

DGF vs. non-DGF	OR	95% CI for OR		p value
		Lower	Upper	
Pre-transplant Hb (g/dL)^#^	0.252	0.159	0.401	<0.001
Donor serum creatinine (mg/dL)^#^	2.038	0.919	4.518	0.08
Donor age (years)	1.002	0.950	1.057	0.37
RBC transfusion (%)	1.609	0.541	4.771	0.39
Dose of rHuEPO (U)/week^#^	1.001	0.998	1.002	0.68
KDPI (%)	0.999	0.974	1.024	0.91

*R^2^ = 0.709; Model (p = 0.02); ^#^logarithmic transformation for statistical analysis; OR: odds ratio; 95% CI: 95% confidence interval; DGF: delayed allograft function; Hb: hemoglobin; RBC transfusion: red blood cell transfusion within first-week post-transplantation; KDPI:* kidney donor profile index.

**Table 3.** Pre-transplant demographic and clinical data

Variables	DGF (n = 120)	Non-DGF (n = 86)	p
**Sex (%)**	F–39 (32.5) M–81 (67.5)	F–37 (43.1) M–49 (56.9)	0.13
**Age (years)**	47 ± 11	49 ± 12	0.17
**Diabetes mellitus (%)**	47 (39.2)	34 (39.5)	0.41
**Hypertension (%)**	23 (19.2)	16 (18.6)	0.44
**MAP (mmHg)**	75.1 ± 10.3	78.3 ± 12.9	0.12
**Creatinine (mg/dL)^#^**	10.7 ± 7.8	9.7 ± 6.9	0.52
**Hb (g/dL)#**	11.8 ± 0.8	12.9 ± 1.0	<0.001
**Fe (µg/dL)**	83.5 ± 29.4	77.5 ± 9.32	0.85
**Transferrin saturation (%)**	42.7 ± 4.1	46.5 ± 12.1	0.91
**Ferritin (µg/L)**	837 ± 229	1344 ± 513	0.43
**Leukocyte (cell/µL)**	8113 ± 3135	7821 ± 3099	0.52
**Platelet (×105 cell/µL)**	1.9 ± 0.53	2.13 ± 0.15	0.65
**PTH pg/mL**	495 + 59.4	405 + 71.4 pg/ml	0.33

Data are reported as number (percentage) or mean and SD. DGF: delayed allograft function; ^#^logarithmic transformation for statistical analysis; MAP: mean arterial pressure; Hb: hemoglobin; Fe: serum iron.

Moreover, KT recipients in the DGF group received higher weekly doses of recombinant human erythropoietin in the pre-transplant period (7483 ± 4185 U/week) than patients from the non-DGF group (6500 ± 4098; p = 0.09). Furthermore, there was a greater need for red blood cell transfusion within the first week after kidney transplantation in the group that progressed with DGF (23 [19.2%]) than in patients who did not progress with DGF (6 [6.9%]; p = 0.01).

We also found that pre-transplant Hb concentration from the kidney recipient was the only variable independently associated with DGF. The model also included donor age, donor serum creatinine, KDPI, a dose of an erythropoiesis-stimulating agent used by the recipient the week before kidney transplantation, and the need for red blood cell transfusion within the first week after kidney transplantation (Table 2).

In addition, there was a higher frequency of kidney recipient patients who used mycophenolate in maintenance immunosuppressive therapy in the DGF group than in the non-DGF group (85 [70.8%]; 49 [58.3%]; p = 0.03).

There was no difference in expanded donors’ frequency between the DGF group and non-DGF group, including kidneys from expanded donors (16 [13,3%]; 9 [10,5%]; p = 0.53). There was no higher acute rejection frequency during the follow-up in the DGF than in non-DGF (26 [21.7%]; 16 [18.6%]; p = 0.59) groups. Out of 206 kidney transplant recipients from deceased donors, forty-three patients (20.9%) developed CAD after 4.7 ± 2.4 years of kidney transplantation. Of these, 32 patients (74.4%) were in the DGF group, and 11 were in the non-DGF group (25.5%; p = 0.02). There was a higher acute rejection frequency in CAD patients (17 [39.5%], 25 [15.3%]; p < 0.001). We also observed that higher erythropoiesis-stimulating agent doses were used before KT (Table 2) on CAD patients. There was no difference in the estimated glomerular filtration rate after three months of kidney transplantation between patients that evolved with and without CAD (43.3 ± 25.1, 48.9 ± 20.7 mL/min; p=0.17, respectively). Nevertheless, a lower estimated glomerular filtration rate was observed after six months of kidney transplantation in patients with CAD than in those without CAD (48.3 ± 25.5, 60.5 ± 20.3 mL/min; p = 0.002). We also found lower Hb concentration after six months of KT in CAD than in non-CAD patients (12.0 ± 2.4, 13.3 ± 2.3 g/dL; p = 0.002) during the study period. We observed a positive correlation between the estimated glomerular filtration rate and the Hb after six months of transplantation (r = 0.49; p < 0.001). There was also an association of Hb concentration six months after kidney transplantation with CAD [OR 0.798, 95% CI: 0.687–0.926; p = 0.003].

Thirty-nine patients (18.9%) evolved with death by any cause after 2.5 ± 0.3 years of deceased donor’s kidney transplantation. There was a lower Hb concentration in both pre-transplant (11.7 ± 0.6, 12.4 ± 1.1 g/dL; p = 0.05) and after six months of kidney transplantation (12.1 ± 2.6 g/dL, 13.2 ± 2.2 g/dL; p = 0.04) in patients dying during the follow-up period. We also observed an association of Hb concentration six months after kidney transplantation with death for any cause in both groups [OR 0.833, 95% CI: 0.705–0.984; p = 0.03]. So, we observed that the frequency of death from any cause was 17.4% in the non-DGF group, while in the DGF group, it was 20% (p = 0.64). DGF and CAD had no association with death for any cause.

## Discussion

The study’s most significant finding was the association between anemia and DGF, CAD, and death from any cause. In addition, there was a lower pre-transplant Hb concentration and higher need for red blood cell transfusion within the first week after kidney transplantation in patients who evolved with DGF. Furthermore, the regression analysis showed an independent association of pre-transplant Hb concentration with DGF.

Thereby, patients with kidney transplant from a deceased donor had a 75% lower chance of developing DGF when the Hb concentration increased by one g/dL above 12 g/dL.

The present study’s results differ from those from another research with kidney transplant recipients published by Na et al.^35^ They observed that pre-transplant Hb concentration was significantly associated with renal allograft function within one year after kidney transplantation. However, there was no association between pre-transplant Hb concentration and DGF. On the other hand, our data are aligned with the results published by Molnar et al.^36^ They reported KFRT-related anemia factors before kidney transplants associated with DGF. Hb concentrations, higher doses of erythropoiesis-stimulating agents, and red blood cell transfusion were related to DGF in their report.

Significantly more patients in the DGF group received mycophenolate as an antiproliferative agent. Antiproliferative drugs also play a role in the pathogenesis of post-transplant anemia^40^. In addition, evidence shows that hyperparathyroidism is associated with anemia in CKD and KFRT^41^. Nevertheless, in the current study, there was no difference in serum levels of basal parathyroid hormone between the patients who evolved with DGF and those who did not evolve with DGF. There was also a higher frequency of acute rejection in patients who evolved with DGF.

The present study showed that patients with CAD had anemia in the pre-transplantation period and required a higher dose of the ESA. This indicates that higher doses of ESA are needed to treat anemia before kidney transplantation. We also found that CAD patients who had lower Hb concentrations after six months of kidney transplant had lower kidney allograft function.

These findings suggest that lower Hb concentration after six months in CAD are due to lower synthesis of EPO, higher inflammation, or retention of uremic solutes due to lower eGFR^3–6,12^. According to other studies, non-surviving patients had lower hemoglobin concentrations six months after kidney transplantation^12,19^. Thus, the current study showed that patients with KFRT and anemia were associated with outcomes after KT, such as DGF, CAD, and death from any cause.

Anemia can contribute to chronic allograft damage by limiting tissue oxygen supply, particularly in the tubulointerstitial area^42,43^. In this way, Cassis et al.^43^ published a research with an animal model in which the effects of erythropoietin post-transplantation were associated with kidney allograft preservation by increased angiogenic factors expression, upregulation of p-Akt, and Bcl-2 anti-apoptotic factors. On the other hand, Elliott et al.^44^ published a meta-analysis about treatment for anemia with ESAs in renal patients where no reduction in DGF or improvement in 1-year graft survival after KT was found.

Furthermore, anemia is common in patients after KT, with a 20–51% prevalence at various time points after transplantation^8,11–14,45^. The current study found that decreased Hb concentration after six months of KT was also associated with CAD and death from any cause. Furthermore, Gafter-Gvili et al.^46^ observed that early post-transplantation anemia in KT recipients was associated with death from any cause, reduced graft survival, and allograft function decline. Moreover, the association with death was related to anemia severity. Our study found an inverse relationship between estimated glomerular filtration rate and Hb concentration after six months of KT. Although Iwamoto et al.^47^ observed a significant correlation between post-transplantation anemia and kidney allograft function, the prognosis for kidney graft function was poorer in patients with Hb levels ≤ 11 g/dL. Okumi et al.^14^ also observed a relationship between reduced kidney graft function and anemia after kidney transplantation.

Finally, regardless of the factors contributing to anemia in kidney disease^3–6,48^, some anemia-related factors were related to outcomes such as DGF, CAD, and death in deceased donor kidney transplantation. These findings can help the interpretation and management of those patients’ outcomes; consequently, the anemia tests could assist nephrologists with decision-making during treatment.

Although intriguing, this study had some limitations. First, this was a retrospective cohort study with a small number of patients with deceased donor kidney transplants. Second, our study was conducted in a single center, and there was no intervention by the researchers. Finally, the study does not rule out the possibility of bias, as anemia is frequent in these patients. Despite these limitations, the current study is consistent with other studies^11,13,14,46^ and raises awareness about the anemia pre- and post-transplantation of deceased donors’ kidneys. Further studies on this topic are necessary to understand the relationship between anemia and kidney transplantation outcomes.

## Conclusion

In conclusion, our study involving kidney transplantation patients from deceased donors found a significant relationship between pre-transplantation anemia and DGF. Besides that, post-transplantation anemia was related to both CAD and death from any cause. Thus, more attention should be paid to anemia pre- and post-kidney transplantation from deceased donors with anemia blood test markers that can facilitate decision making for treatment complications.

## Data Availability

The data that support the findings of this study are available in figshare (10.6084/m9.figshare. 20563206).
